# Facilitators and barriers to effective scale-up of an evidence-based multilevel HIV prevention intervention

**DOI:** 10.1186/s13012-015-0216-2

**Published:** 2015-04-17

**Authors:** Susan M Kegeles, Gregory Rebchook, Scott Tebbetts, Emily Arnold, The TRIP Team

**Affiliations:** Center for AIDS Prevention Studies, University of California, 550 16th Street, 3rd Floor, San Francisco, 94158-2549, CA USA

**Keywords:** Translating research into practice, Translational science, HIV prevention interventions, Gay men, Implementation science, Young gay men, Young men who have sex with men, Structural interventions, Community level interventions, Community mobilization, Fidelity, Community-based participatory research

## Abstract

**Background:**

Since the scale-up of HIV/AIDS prevention evidence-based interventions (EBIs) has not been simple, it is important to examine processes that occur in the translation of the EBIs into practice that affect successful implementation. The goal of this paper is to examine facilitators and barriers to effective implementation that arose among 72 community-based organizations as they moved into practice a multilevel HIV prevention intervention EBI, the Mpowerment Project, for young gay and bisexual men.

**Methods:**

CBOs that were implementing the Mpowerment Project participated in this study and were assessed at baseline, and 6-months, 1 year, and 2 years post-baseline. Semi-structured telephone interviews were conducted separately with individuals at each CBO. Study data came from 647 semi-structured interviews and extensive notes and commentaries from technical assistance providers. Framework Analysis guided the analytic process. Barriers and facilitators to implementation was the overarching thematic framework used across all the cases in our analysis.

**Results:**

Thirteen themes emerged regarding factors that influence the successful implementation of the MP. These were organized into three overarching themes: HIV Prevention System Factors, Community Factors, and Intervention Factors. The entire HIV Prevention System, including coordinators, supervisors, executive directors, funders, and national HIV prevention policies, all influenced implementation success. Other Prevention System Factors that affected the effective translation of the EBI into practice include Knowledge About Intervention, Belief in the Efficacy of the Intervention, Desire to Change Existing Prevention Approach, Planning for Intervention *Before* Implementation, Accountability, Appropriateness of Individuals for Coordinator Positions, Evaluation of Intervention, and Organizational Stability. Community Factors included Geography and Sociopolitical Climate. Intervention Factors included Intervention Characteristics and Adaptation Issues.

**Conclusions:**

The entire ecological system in which an EBI occurs affects implementation. It is imperative to focus capacity-building efforts on getting individuals at different levels of the HIV Prevention System into alignment regarding understanding and believing in the program’s goals and methods. For a Prevention Support System to be maximally useful, it must address facilitators or barriers to implementation, address the right people, and use modalities to convey information that are acceptable for users of the system.

## Background

The field of Implementation Science is rapidly expanding as research into the creation of evidence-based interventions has yielded innovative approaches to ameliorate various problems, particularly adverse health issues. Considerable research has focused on the dissemination of information about evidence-based interventions (EBIs), since they cannot be adopted if potential implementing organizations are unaware of them. Other research has focused on various aspects of implementation, with the understanding that once organizations have decided to adopt an EBI, the program needs to be moved into effective practice. As the field advances, numerous researchers are developing dissemination and implementation (D&I) models. A recent paper analyzed 61 D&I models and found that most models emphasized dissemination rather than implementation of interventions: 27 models focused on dissemination, 17 models emphasized both equally, and only 17 focused on implementation issues. Of this latter group, only 12 models entirely focused on implementation [1].

The level of implementation achieved is an important determinant of effectiveness outcomes. Since poor implementation outcomes can impact effectiveness outcomes, it is critical to discriminate between implementation outcomes (Are they doing the program as intended?) and effectiveness outcomes (Is it resulting in good outcomes?) [2]. Implementation in this paper means, “the use of strategies to adopt and integrate evidence-based health interventions and change practice patterns within specific settings” ([3] p.424). When evidence-based HIV prevention interventions are implemented in the “real world,” they can achieve effects similar to those in the original research [4-7]. But how EBIs are implemented, including the intervention’s core elements (required project components that embody the intervention’s theory and internal logic and most likely produce the intervention’s main effects [8,9]), has a substantial impact on their effectiveness and sustainability over time [10]. Multiple implementation studies in the HIV prevention field have found that organizations often fail to implement all required core elements or adapt them significantly [11-14].

Although some evidence-based HIV prevention interventions have been widely adopted [15-19], including the intervention studied in this paper, the Mpowerment Project (MP), the scale-up of HIV/AIDS EBIs has not been simple and the “implementation gap,” the translation of scientific knowledge to effective and efficient program implementation, has received increasing attention [20,21]. The MP is a cost-effective HIV prevention intervention EBI for young gay/bisexual men (YGBM) that was tested in randomized, controlled trials and found to be effective in reducing rates of unprotected anal intercourse [22-24]. Widespread dissemination of the MP occurred because the CDC promoted it and other EBIs to health departments and community-based organizations (CBOs) as part of the “DEBI” (Diffusion of Effective Behavioral Interventions) project that sought to move HIV-prevention related EBIs into practice [25]. The present research focuses on implementation of the MP by CBOs that obtained funding to implement it. Examining implementation of the MP is important both because it focuses on YGBM, a population that remains at high risk for HIV transmission [26], and because it is a multilevel HIV prevention approach, which has been identified as critical for HIV prevention [27] in the US National HIV/AIDS Strategy [28] and the CDC’s High-Impact Prevention efforts [29].

The overall goal of the TRIP (Translating Research into Practice) Study was to determine if providing innovation-specific capacity building assistance improved the implementation of the MP by CBOs. Research to practice models are based on the paradigm that scientists conceptualize and establish an intervention’s efficacy, and then CBOs are expected to implement it [30,31]. This transfer of knowledge approach, from scientists to community, is in contrast with community-centered models, which are based on a paradigm that emphasizes improving practice within the context of the community while considering the resources and needs of the community. This means that capacity building assistance strives to help CBOs adapt interventions to the community context [30]. TRIP’s capacity-building approach was a blend of the two models. Our intent was for organizations to implement the MP as operationalized in the efficacy trials, including using community-based participatory methods, while also adapting the intervention to their communities in ways that preserve the core elements and conceptual underpinnings of the program (which we formulated into MP’s “guiding principles”; see Table 1). In this paper “successful” or “effective” implementation (used interchangeably) mean the extent to which intervention fidelity is maintained while adapting the MP for specific communities. The underlying assumption is that fidelity to the intervention is most likely to result in outcomes that approximate those achieved in the original trials.

**Table 1:** **Overview of the Mpowerment Project**

**Structure and principles underlying MP**	**Description of MP**
*Overview of MP*	• Community-level, community mobilization intervention targeting all YGBM in a community
	• Focuses on structural-level changes, including altering YGBM’s social milieu through development of a YGBM’s community that supports each other regarding sexual risk reduction and frequent HIV testing, and by providing a community space where men can find supportive, caring community in a safe environment where they encounter social norms supportive of sexual risk reduction and frequent HIV testing [32]
	• Focuses on social-level issues, including norms and social support about safer sex and HIV testing
	• Focuses on interpersonal factors, such as communication and sexual negotiation skill-building, and boyfriend issues
	• Focuses on individual-level factors, such as clarifying misperceptions of what is safe and unsafe, why regular and frequent HIV testing is important, and internalized homonegativity
	• MP initially only focused on sexual risk reduction, but now is also used to promote regular and frequent HIV testing and is being adapted to focus on the HIV Continuum of Care [33]
*Guiding principles*	• 6 principles below, based on formative research and theories of social and behavioral change, underlie MP
	• Has been intended, since initial development, that the MP is adapted for and by each community for their populations, settings, and cultural issues following careful consideration of the core elements and guiding principles
Social focus	• HIV prevention is not very salient to YGBM, they are not drawn to HIV prevention programs
	• Must relate risk reduction and HIV testing to the satisfaction of more compelling needs, such as the development of new friends and social networks, enjoyment of social interactions, and enhancement of self-esteem
Community-building	• Primary goal of the MP: to create healthy friendship and social support networks
	• Creates settings where YGBM can express their identities, form positive linkages with similar others, and draw support and band together to take action on issues of importance to them
Peer-based	• Seeks to mobilize men to support and encourage their peers about having safer sex and getting tested for HIV regularly
	• Peers used as change agents because they exert tremendous influence at this life stage of life and are credible
Empowerment philosophy	• Designed to serve an empowering function within the YGBM community
	• Behavior change is more lasting when people are actively involved in finding and implementing solutions to their problems
	• Providing mechanism for designing and running the intervention activities fosters sense of personal commitment to and ownership of prevention activities and messages
Diffusion of innovations	• Develops process by which YGBM actively communicate with each other about and encourage each other to practice safer sex and get tested for HIV regularly (new behavioral practices) so they become mutually accepted norms
Gay, sex, and ethnic/racial group positive	• MP attempts to enrich and strengthen YGBM’s pride in being gay or same gender loving and nurture their exploration and celebration of their sexuality
	• For MSM of color, the project nurtures pride in being of one’s ethnic/racial group
*Core elements*	• 7 core elements, described below, work synergistically with the guiding principles and set in motion an ever-widening diffusion process through which young men communicate with and encourage each other about sexual risk reduction and regular, frequent HIV testing
Coordinators	• Paid CBO staff, typically young gay/bisexual men from the community, who facilitate the project and coordinate its activities
Core group	• 10 to 20 young men from major subgroups in the community and the coordinators
	• The decision-makers for the project and design and carry out all project activities
Formal outreach	• Teams of YGBM go to locations frequented by other YGBM to promote safer sex and testing in engaging and interactive ways
	• Distribute appealing literature on testing and HIV risk reduction (developed in-house) and condoms and lubricants
	• Also create social outreach events to attract YGBM from different subgroups, at which to promote safer sex and testing
Informal outreach	• Men encourage friends to be safe sexually, attend project activities, and join project
	• M-group participants are trained and motivated to conduct informal outreach
M-groups	• Peer-led, 3-h meetings of 8 to 10 young men
	• Uses skills-building exercises to address factors contributing to unsafe sex or infrequent HIV testing among the men
Publicity	• Publicizes and attracts men to project
	• By word of mouth, via social networking and websites, through the distribution of promotional materials at venues attracting YGBM, and through articles and advertisements in gay media

The TRIP study, a longitudinal project described previously [14,34], had two primary objectives: (1) to determine if an intervention for CBOs implementing the MP, called the Mpowerment Project Technology Exchange System (MPTES), would result in increases in MP fidelity across the CBOs over time, and (2) to gain an understanding of the issues CBOs experienced while running the EBI, specifically focusing on barriers and facilitators to effective implementation.

The MPTES is an integrated system of training, ongoing technical assistance (TA), implementation materials (manuals and videos), and web-based services, and collaboration with CBOs was used extensively in its development. The MPTES is based on social learning theory [35], diffusion of innovations [36], and theories and approaches to adult education [37]. Since there is a paucity of research or theory about the efficacy of different approaches to capacity building in order to improve intervention fidelity [1,3], the MPTES primarily focused on issues that had arisen in our previous work with CBOs that had contacted us for assistance [34]. The Interactive Systems Framework for Dissemination and Implementation has been developed as a heuristic to conceptualize the systems involved with moving an innovative program into practice [38]. A Prevention Support System is a key component that involves the provision of assistance to CBOs implementing an innovation. The MPTES is a Prevention Support System, and primarily targeted frontline staff who carry out the daily tasks of implementing the MP (called “coordinators”), although some information was targeted at supervisors.

As the primary organization that provided information and training to CBOs on the MP, we had access to all organizations seeking information on the MP. We provided the MPTES proactively to CBOs, and assessed each organization for two years. The goal of this paper is to examine the barriers and facilitators to effective implementation of the MP by CBOs that arose while the organizations were running the intervention during this study. See Table 1 for a brief overview of the MP.

## Methods

All but 2 of the 74 CBOs implementing the MP agreed to participate. Organizations were recruited into the study through telephone calls and letters. We first obtained executive directors’ (EDs) or HIV prevention directors’ consent for the agency to participate in interviews. Then individuals we interviewed provided their own consent to participate. Interviews were conducted at baseline, when each CBO was recruited into the study and after which TA was provided; 6-months post-baseline, after which CBOs were provided with more TA; one year post-baseline, after which CBOs were offered more TA; and two years post-baseline. Since logistically the interviews at all participating CBOs could not be conducted simultaneously, we used rolling enrollment with new CBOs entering into the study continually over time until no other CBOs implementing the MP could be identified. Semi-structured telephone interviews were conducted separately with 2–4 individuals at each CBO, including the coordinators; their supervisors, who were typically the agencies’ HIV prevention directors; and 1–2 core group members (YGBM volunteers who serve as decision-makers to MP projects). Due to high job turnover, only a third of participants were interviewed multiple times over the two-year data collection period for each CBO. Participants were asked to locate an area at work where they could participate in the interviews while maintaining privacy. Participants were compensated $25 per interview, and the University of California San Francisco’s institutional review board approved the research protocol.

Data for this paper came from two sources: telephone interviews and extensive written TA fieldnotes (see Table 2). Barriers and facilitators to implementation was the overarching thematic framework used across the data analysis. Principles of Framework Analysis [39] guided the analytic process, allowing the research team to use the predetermined thematic framework, as well as to capture emergent codes within the data. The team met biweekly to discuss the data, note emergent themes, and refine the analytic process. After reviewing approximately 150 interview summaries and TA fieldnotes, the coding schema was operationalized by elaborating on *apriori* codes, emergent codes, and their subcodes, which were the most prominent organizing themes in the data. The first author completed extensive memo writing after each analysis session. The codebook was finalized after reviewing approximately 200 interview summaries and fieldnotes, and then applied across the dataset. Analysts continued to meet regularly, discussing any discrepancies that emerged during data collection or coding, and discussing particular illustrative cases in great detail to further understand the theoretical processes emergent in the data, and further refine how the codes related to one another.

**Table 2:** **Data sources, data analysis and quality control methods used**

**Data source**	**Topics noted**	**Quality control**	**Methodological approach**
Semi-structured telephone interviews (N = 647)		Interviewer:	For both sources of data:
	• How each core element is being implemented	• Trained to take extensive notes, including key verbatim phrases to record content of each interview	• Investigators regularly reviewed interviewer’s and TA providers’ interview summaries and field notes
	• Adaptations made to the core elements	• Cleaned each summary note, making sure team had data on each relevant topic in interview guide	• Bi-weekly meetings addressed TA and field notes
	• Rationale for adaptation	• Indicated verbatim phrases	• Discussions focused on barriers and facilitators to effective implementation, as well as how to address them in TA
	• Problems encountered in implementation	• Ensured accurate record of interview content and interview conditions (i.e., level of rapport, apparent distractions, general level of flow for each interview)	• Field notes, summaries, and analytical codes applied to relevant sections of each summary note were entered into qualitative database
	• Approaches used to overcome challenges		
Extensive field notes and commentary		TA providers:	
	• Barriers and facilitators to implementation fidelity	• Had been coordinators in prior efficacy trials, and were extensively trained and supervised in previous research	
	• Problems encountered in implementation	• Thus had clear understanding of fidelity to original implementation methods	
	• Approaches used to overcome challenges	• Jotted extensive notes during all TA sessions (delivered by telephone and/or e-mail)	
		• Subsequently created detailed commentary about each TA session, operationalized as fieldnotes	
		• Were trained on the study domains (barriers and facilitators to intervention fidelity) to ensure recording of relevant data when topics of interest arose during TA	
		• Used template for TA fieldnotes, which contained headings for each relevant study topic where notes were taken	

## Results

The CBOs were quite diverse (see Table 3). They served different ethnic/racial groups of YGBM, were located across the US, and the size of their budgets for the MP varied substantially. Thirteen themes emerged regarding influences on the successful implementation of the MP, which were organized into three overarching themes: HIV Prevention System Factors, Community Factors, and Intervention Factors (see Table 4). Examples of facilitators and barriers to successful implementation are included in the descriptions and verbatim quotes from the participants are presented in Table 4.

**Table 3:** **Characteristics of the 72 community-based organizations in the study**

**Types of characteristics**	**Breakdown of characteristics**	**Data on participating CBOs**
Type of organization	AIDS service organization	75.5%
	Lesbian/gay/bisexual/transgender center	2.0%
	Other CBO	10.2%
	Local health department	4.1%
	University	2.0%
	Other health care agency	4.1%
	Foundation/funder	2.0%
Number of full-time equivalent positions at organization	Total at agency	Range: .50 to 750
		Mean = 60.5
		Median = 24.0
	Total in HIV prevention	Range: .50 to 100
		Mean = 9.4
		Median = 6.0
Overall organization budget/year	Range	less than $250,000 to over $2,000,000
	Median category	500,000 to 1,000,000
Primary focus of organization	HIV/AIDS	55.1%
	Other	44.9%
Community population size	Range	30,000 to 11,000,000
	Mean population	1,259,000
	Median population	600,000
States where project located	31 states, plus the District of Columbia and Puerto Rico

**Table 4:** **Barriers and facilitators to successful implementation of the empowerment project**

**Overarching issues**	**Related themes**	**Illustrative examples**	**Quotes from notes interviewer or TA providers wrote**
HIV prevention system factors	The entire HIV prevention system affects intervention implementation	Coordinators, supervisors, EDs, funders, and national HIV prevention policies all greatly influenced implementation. For example, in numerous situations, funders would not financially support a core element (see quote). Additionally, coordinators were sometimes eager to implement the intervention with fidelity, but issues with CBO management would adversely affect implementation. For example, one of the MP’s guiding principles (see Table 1) is that since most YGBM do not seek HIV prevention services and often intentionally avoid AIDS organizations, HIV prevention must incorporate a social focus to attract them. There were a number of situations in which a coordinator and core group developed ideas for an event but were stopped from enacting them because CBO management did not support using social events in HIV prevention efforts or disliked the event. Stopping the core group from enacting their ideas undermined their sense of ownership of the project, caused them to lose interest in volunteering, and may have resulted in their not sharing MP’s prevention messages within their social networks. HIV prevention policy initiatives also negatively affected implementation of the MP. To obtain funding from the CDC or state health departments, CBOs were usually required to implement a DEBI intervention, i.e., an intervention that had been shown through rigorous research to have evidence of its effectiveness. Initial resistance to DEBI cast a shadow on the MP and resulted in some CBOs distrusting the MP as suitable for their populations, while others were reluctant even to learn about the MP through using the MPTES. Antagonism towards DEBI often had to be overcome in order to develop rapport with those receiving TA. A second HIV prevention policy that stymied implementation was the push for CBOs to conduct more HIV testing, since the resources for this generally came from redirecting funds away from the EBI. The third policy-related issue that affected implementation was the requirement that all publicity or safer sex materials that were even partially supported by the CDC had to be reviewed by a local Program Review Committee. Since some committees rejected materials that were sex- or gay-positive, some CBOs’ management would self-censor the materials before review.	“When he [the coordinator] met with XXX, the guy from the health department…they don’t want to pay for him to do activities that are associated with social events [a major Core Element of the intervention]…they only want to pay him to do the M-groups and the outreach.”
	Knowledge about intervention	Funders, CBO management and staff needed to know about MP to implement with success. Funders who lacked an understanding of the program often developed contracts that contained unrelated “deliverables,” objectives that the CBO was required to achieve (see quote). The CBOs then had to choose between fulfilling their contractual obligations or implementing the MP with fidelity. For example, weekly core group meetings and regular social events are necessary to implement MP with fidelity, but many contracts did not include core group meetings or social events in them, and instead required an unrealistic number of M-groups resulting in an unsuccessful MP implementation. CBOs that were funded under these circumstances were in a dilemma about how to implement the intervention with fidelity—needing to put a “round” community-level intervention into a “square” group- or individual-level oriented contract. Knowledge about the program’s core elements and guiding principles was also important for CBO management and staff alike. For example, some coordinators did not understand the guiding principle that the project should facilitate the empowerment of YGBM, and wanted to make all decisions for the project themselves instead of supporting the core group to analyze the issues facing their community and determine solutions they would enact. Similarly, supervisors were helpful when they understood the intervention well enough to assist coordinators to prioritize tasks, or problem-solve issues that arose. However, some supervisors did not understand the intervention, and a few expressed beliefs that it was unnecessary for them to learn about it since the MP is for YGBM, and YGBM could therefore conduct the program with little supervision. Likewise, EDs varied in their understanding of the intervention, and some felt uncomfortable with its innovative aspects. For example, some EDs disliked YGBM being the decision-makers for the program because they worried that the young men would make decisions that could harm the organization and/or its reputation.	“The biggest difference between the way they do the model is that they have to follow the contract that the county lays out, which takes it far from the intervention…they have to do tons of things that are different from the model.”
	Belief in efficacy of intervention	Coordinators who believed that the MP would be effective were most enthusiastic about implementing the MP with fidelity. For example, some coordinators believed that the MP would not work in large urban settings, and approached the intervention with defeatist attitudes that became a self-fulfilling prophecy (see quote), whereas coordinators working with similar populations and approached the intervention with enthusiasm and creativity appropriately adapted the MP to reach their communities successfully. Beliefs about the efficacy of the intervention were especially important in projects targeting young ethnic/racial minority men.	“They [the core group] don’t plan anything as a group…the curriculum is ideal but unrealistic for real life as they know it…people are busy…so the coordinators do the events…she thinks that that part of the program, the core group planning stuff, is a joke and needs to be changed…maybe in a place where there is nothing to do but pick your nose, but in [a large city], it isn’t going to happen.”
	Desire to change agency’s existing prevention approach	Challenges occurred when staff did not want to change what they were doing or didn’t care that much about changing what they were already doing (see quote).	“They had been trying to get it [MP] going…or something like it….they had a lot of people coming, but it wasn’t focused…it didn’t look like the model…the agency didn’t really care**.** They do outreach once a week at the clubs, and they do referrals for [HIV] testing and STD testing… they go through and give out safer sex kits to anyone who will take them from them....[but] they haven’t redeveloped the kits for Mpowerment.”
	Planning for intervention *Before* implementation	MP worked best when CBO management planned ahead to secure proper space and staff (see quote). When pre-implementation planning did not occur, poor decisions were often made that had long-term deleterious effects. CBOs often hired someone outside the organization to write the grant proposal, and did not analyze what would be needed for the program to function adequately or if the organization was truly poised to implement the intervention if they were funded. Often organizations only learn one month beforehand that they are funded, which does not facilitate careful planning for implementation.	“The room they’ve ended up using is quite sterile and housed within the AIDS project.”
	Accountability for work	A lack of accountability affected how tasks were performed and deliverables achieved. Sometimes coordinators were largely left on their own and did not necessarily follow through on tasks. Besides the work not getting done, when this occurred core group members and other volunteers wondered why they should work for free when paid staff did not, and the volunteers would drift away from the program. A few supervisors were aware that the coordinators were underperforming but did little about it. Similarly, occasionally EDs did not hold their supervisors responsible for their staff members’ productivity, and funders did not hold the CBOs accountable for their work. Although deliverables that were part of contractual agreements were not achieved, sometimes there seemed to be little attention given to this deficit. In contrast, when staff were held accountable for their work, implementation went far more effectively.	“When she [the supervisor] goes to talk to [the ED] and she tells him about stuff that is going on with the project, he just sits there and nods…he doesn’t provide any direction for her and is more apt to err on the side of supporting the coordinators rather than questioning if they are a good fit or not…this makes it difficult for her…when she thinks that they may need to be fired.”
	Appropriateness and capacity of individuals for coordinator positions	It was sometimes difficult for agencies to recruit and hire appropriate coordinators. In contrast, projects were typically run well when the coordinators were YGBM who were outgoing, analytical, impassioned about HIV prevention, and hardworking; were enmeshed in gay community social networks; and had or obtained a variety of skills (e.g., to create databases, facilitate groups, create a publicity plan).	“She hired someone on recently who has a marketing background…who is tuned in with what is attractive and fun… but when it comes to promoting something…he is like ‘I don’t know, I don’t know…I don’t know’…she thinks that even with the training, and with the weekly meetings…he feels a bit overwhelmed…he is good at databases and stuff like that…but publicity, marketing, recruitment…it is very hard.”
	Evaluation of intervention’s functioning	Staff must be willing to evaluate the program in order to improve the program.	“And now they are starting to think about who they aren’t reaching…who are the people that you aren’t reaching out to…he doesn’t want it to be just about handing out flyers…he spends a lot of time getting the guys to reflect on what they are doing with the project.”
	Organizational stability	When the CBO experiences financial crises it is difficult to implement the MP. One organization, for example, showed high fidelity in implementation when it had stable funding, but considerable changes occurred at the CBO over time. The ED left, and the new one was not supportive of the MP. Then the organization went bankrupt.	“He [the funder] thinks that the project needs to go to a different organization…the agency isn’t stable enough.”
Community factors	Geography	The size of the city and its proximity to a gay magnet city affected the ability of coordinators to recruit and retain YGBM in the intervention. Small communities sometimes presented challenges to implementation because there could be too few men to mobilize and build a YGBM community. Some CBOs dealt with this by expanding the age range of the project participants to include more men. Small communities that included a large university, however, usually implemented MP well. Implementing the intervention in rural areas where there were insufficient young men was often challenging. Implementation in large cities varied. Although larger cities would seem to provide more pre-existing social opportunities, this is not always the case for YGBM. Often implementation worked well, as MP filled the niche of social activities for YGBM. When the project focused on one ethnic/racial group in large urban environments, it seemed easier to implement successfully than when the project attempted to reach all YGBM, again because it filled a niche. Another community issue that impacted implementation was proximity to a “gay magnet city.” It was difficult to attract men to the project when it was implemented near such a locale since often they would rather go to the city rather than stay in their own community to attend MP outreach events.	“Trying to compete with San Francisco [to attract YGBM] wasn’t working” [respondent’s CBO was located in a county only a few miles away].
	Sociopolitical context	It can be challenging to implement some core elements with fidelity because of hostile responses in conservative areas.	“Publicity was hard because they couldn’t even have a website that was geared toward gay men because the county is so conservative and the funder [the county health department] didn’t want to risk creating a commotion.”
Intervention factors	Intervention characteristics	As a multilevel, multicomponent, community-wide program, the MP is complex and requires significant staff time and funding. Programs with insufficient funding were more likely to flounder as they decided how frequently to implement the core elements since they could not follow our recommendations about this. CBOs and funders alike were uncertain how to implement the MP since they could not afford to have large outreach events frequently or much of a publicity campaign, for example. They wanted more guidance about how to adapt the project with less funding.	“When he went to Atlanta [to a conference], he saw [a presenter connected with the MP research] and he talked to him and went to his presentation, and at the time they didn’t like the [intervention] because they thought that they [the researchers] had all this money to do it and it wasn’t the real world…because they were talking to CBOs that were working on shoestring budgets and they don’t have lots of money for training and planning like they [the researchers] had.”
	Adaptation issues	CBOs wanted guidance about adapting the MP to diverse locales and young gay Black or Latino populations to ensure fidelity with the guiding principles. For example, a number of projects added building life skills to the core group; used balls (performance events) as social outreach events; added mental health counseling or linkages to other services (e.g., HIV testing, linkage to care for HIV-positive men, emergency housing); paid stipends to core group members; and altered the structure of the core group, all of which could be done in accordance with the guiding principles. Other adaptations, however, did not contribute to successful implementation. For example, several CBOs changed M-groups into ongoing discussion groups. Since new young men were not recruited for these groups, informal outreach into new social networks was limited.	“The question of fidelity is something that they talked about a lot…the boxed [DEBI] interventions are great, but what people really need is more TA about how to effectively adapt these interventions while retaining the theoretical core. They [the agency] needs to build their capacity to understand the internal logic of the M-group piece of the intervention so that they can say ‘here is the logic of this activity, and the behavior it is seeking to address…here is our target population for this intervention…how do we change M-groups for this target population while retaining fidelity to the original design?’”

### HIV prevention system factors

#### The entire HIV prevention system affects intervention implementation

Coordinators, supervisors, executive directors (EDs), program funders, and national HIV prevention policies all greatly influenced implementation success, and program implementation suffered when there was not alignment in views about the intervention among these entities. For example, sometimes coordinators wanted to implement the MP with fidelity, whereas supervisors or EDs did not see the value of doing so as they distrusted a guiding principle (e.g., using social events to attract YGBM; see Table 4). Funders sometimes strongly positively or negatively affected implementation. In numerous situations, funders would not financially support a core element (see Table 4), but at other situations funders would push agencies to implement with fidelity by urging them to implement dropped core elements. Having coordinators, CBO management, and funders in alignment resulted in the most effective implementation. National HIV prevention policies that required CBOs to adopt interventions that had been shown to have evidence of effectiveness, such as MP, sometimes negatively impacted implementation when they were met with resistance by CBO staff (see Table 4).

#### Knowledge about the intervention

Staff at all levels of the CBOs who had a deeper understanding of the intervention were substantially more able to implement it with fidelity than were those who did not fully understand it. A lack of knowledge about the intervention affected analysis of the community, subsequent solutions, prioritization of tasks by front line staff and the abandonment of core elements and guiding principles originally designed to support the intervention (see Table 4).

It was also important for funders to understand the program’s core elements and guiding principles. Those who understood the intervention were more likely to develop contractual language that supported implementation with fidelity, whereas funders who lacked an understanding of the program often developed contracts that contained unrelated “deliverables” that the CBO was required to achieve (see Table 4).

#### Belief in the efficacy of the intervention

To run the intervention with fidelity, coordinators, supervisors, EDs, and funders all needed to believe that implementing the EBI would lead to sexual risk reduction with their target population. This was especially true for coordinators, as those who believed that the MP would be effective for their group were most enthusiastic about implementing the MP with fidelity (see Table 4).

#### Desire to change agency’s existing prevention approach

Frontline staff and management who felt that their previous HIV prevention strategies were ineffective were far more willing to implement the MP with fidelity, as were staff who saw the value of implementing EBIs. But institutional sources of resistance to change sometimes stymied the efficient adoption of the MP. Staff were sometimes slow to adopt the intervention out of inertia (see Table 4). While they did not necessarily believe what they had been doing was effective, they were not necessarily desirous of change. Other times staff believed they saw a similarity between the MP and their current strategy, and simply relabeled their previous program the MP without making changes. Some CBOs adopted the MP solely because funding to do so was available, but had little desire to work with YGBM. This did not bode well for implementation.

#### Planning for intervention before implementation

An important theme that emerged was that project implementation was more efficient and effective when CBO management, particularly EDs, planned ahead to implement the MP than when they did not have such preparation. When planning did not occur before CBOs received the funding, it resulted in management who did not recognize the need for a space or sufficient and appropriate staff, did not budget to fully implement all of the core elements, or did not make necessary policy changes at the CBO (e.g., allowing staff to work after business hours; see Table 4). Often organizations only learn a short time beforehand that they are funded, which does not facilitate careful planning for implementation.

#### Accountability for work

Coordinators need to accomplish many tasks for the program to function given its multiple core elements, and those who put effort into their jobs were substantially more effective at implementing the intervention than were men who put in little effort. CBOs varied considerably in the extent to which they held coordinators accountable for their work (see Table 4).

#### Capacity and appropriateness of individuals for coordinator positions

Coordinators could make or break the implementation of the MP. Successful coordinators attracted YGBM to the project, were the starting point of diffusion into YGBM communities, and helped core groups develop creative, appealing, and innovative activities. But CBO management were not always mindful about the characteristics and skills needed to effectively lead this project, and instead, hired men who were already working at the CBO who fit the demographics (male, gay, young), and assumed they could run it. Hence, CBOs sometimes hired coordinators who alienated others, were social isolates, had poor interpersonal skills, or lacked the capacity to run the program (see Table 4).

#### Evaluation of intervention’s functioning

Although ongoing reflection and evaluation of each core element’s implementation is necessary, some coordinators never critically assessed how their program was functioning, despite having ready-made, user-friendly, simple process evaluation materials in the implementation manual to use. They did not analyze if the program was achieving its objectives, if it was reaching new YGBM and social networks, and what, if anything, about their program needed to change. Frontline staff and CBO management who engaged in critical, ongoing analysis of program functioning were considerably more effective at implementing the intervention.

#### Organizational stability

When CBOs experience constant flux/financial crises it was difficult to implement the MP. It was clear that CBOs were unlikely to implement the intervention effectively when they were struggling to remain solvent or are going through substantial staff turnover.

### Community factors

#### Geography

Where the MP was implemented affected implementation. Population size was important. The MP was originally developed for mid-sized cities (populations ranged from 100,000-1.5 million), where it attracted YGBM because it filled a gap: young men needed social opportunities separate from bars, clubs, and “cruising” places. The MP fills this gap in many locales and attracts young men by providing diverse social opportunities in a safe environment. Small communities, rural areas and communities within close range of a “gay magnet city” experienced difficulty attracting men (see Table 4).

#### Sociopolitical context

The degree to which the sociopolitical environment marginalized YGBM was another community issue that affected implementation. It can be challenging to implement some core elements with fidelity because of hostile responses in a conservative area, and staff often needed to spend considerable time dealing with such issues. For example, it can be challenging to find a project space because some landlords did not want to rent their space for a YGBM or HIV project.

### Intervention factors

#### Intervention characteristics

Three intervention characteristics were barriers to implementation. The first barrier stemmed from MP being a multilevel, multicomponent, community-wide program and therefore complex, requiring significant staff time and effort. Many CBOs were underfunded, which resulted in insufficient staff to accomplish MP’s activities.

The second MP characteristic that was sometimes a barrier to implementation is that the intervention is not highly scripted, except for the M-groups, and the implementing agency has to decide precisely how to operationalize it. Implementing the MP effectively, which can include adapting it, requires understanding the purpose of each core element and guiding principle, and how they all relate to each other. However, many CBO coordinators and management never or rarely looked at any of the MPTES materials, did not attend a training on the intervention, and hence, did not understand the intervention well [34].

The third challenging MP characteristic is the need to use empowering, participatory methods. It can be difficult for the coordinators to learn how to draw out, listen, and incorporate the views of participants rather than simply directing the program themselves. Yet simply running the program without YGBM’s involvement has various adverse consequences, such as poor attendance at activities and little reach into diverse social networks.

#### Adaptation issues

Many organizations felt that adaptation of the intervention was essential for the MP to work in their community. Some coordinators understood how to adapt the MP using the guiding principles and retaining the core elements, and therefore created adaptations that were entirely in alignment with them. However, other adaptations did not contribute to successful implementation (see Table 4). Occasionally CBOs intentionally dropped core elements entirely as a part of adapting the intervention. Other times, however, core elements were dropped simply because they were overlooked. Other organizations wanted specific guidance about adapting the MP for their populations, and did not want to make adaptations themselves.

### Importance of the themes for implementation success

The themes subsumed under HIV Prevention System factors and Intervention factors affected implementation far more frequently than did Community factors. The most frequent themes that arose concerned the coordinators’ capacity and appropriateness to serve as frontline staff for the MP; that the MP is a multicomponent intervention and thus requires adequate resources; planning for the intervention before implementation; and adaptation. Themes that occurred less often than others were staff members’ reluctance to change existing programming, and geography, meaning proximity to gay magnet areas.

It was difficult to determine if CBO characteristics were related to the effectiveness of implementation, other than sometimes being “insufficient” for implementation (e.g., very small organizations often could not implement the MP effectively because of insufficient resources). Yet a program associated with a university and which had few staff was implemented well, because their focus was on a limited group (students), whereas large organizations did not necessarily excel in their implementation since they sometimes had inertia in implementing a new approach or the management simply did not trust YGBM to make decisions, thus alienating volunteers and their staff. In general it seemed that AIDS organizations were able to implement the MP more effectively than non-AIDS organizations, although this varied as indicated above with the case of the university. Yet there was a considerable range of organizations that were the non-AIDS organizations and it does not necessarily make sense to lump them together in order to contrast them with AIDS organizations.

## Discussion

This is one the first studies of an HIV prevention intervention that has looked at implementation issues across many CBOs, longitudinally, and in-depth; many implementation researchers have bemoaned the infrequency of this kind of research [3,40]. We found that the entire HIV Prevention System, including frontline staff, CBO management (both supervisors and EDs), funders, and HIV national policies strongly impacted the extent to which the program was moved into practice successfully. The most successful implementations occurred when the HIV Prevention System was in alignment with respect to the frontline staff, management, and funders. We had not anticipated that funders would be an important factor, such as when they pushed their CBOs to implement core elements with greater fidelity, or developed contracts to implement the MP that did not reflect the intervention. Similar to findings reported in other studies, national HIV prevention policies, social determinants, and the community in which the program was implemented also impacted implementation, as did intervention characteristics [41,42]. Additionally, many issues revealed in this study correspond with facilitators and barriers to implementation that have been identified in translating research into practice outside of HIV/AIDS [30,40,43].

**Figure 1:**
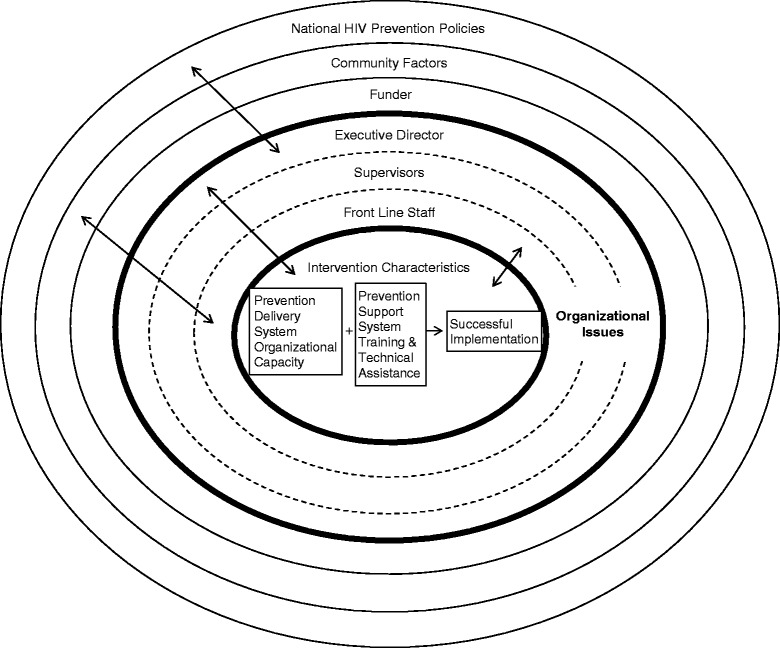
Framework for understanding the forces impacting successful implementation.

The results obtained in this study are consistent with the Consolidated Framework for Implementation Research (CFIR) [44], which identifies five domains important to consider when studying implementation. These domains include the intervention itself, outer setting characteristics, inner setting characteristics, individuals, and process. Each of the five domains is comprised of a set of constructs that are believed to positively or negatively influence implementation. A multilevel ecological perspective, including CFIR, is necessary for understanding successful implementation, as others have proposed [31,45-48]. However, with an eye towards the development of effective Prevention Support Systems, we propose some changes. Figure 1 is a heuristic to capture these multiple levels, and is adapted from Durlak and DuPre [48].

The frontline staff, supervisors, and EDs should not be subsumed under general “provider characteristics” or “organizational factors”, since how they function vis-à-vis the intervention differs substantially, and each uniquely affects the implementation process. This is indicated through the dashes shown in the figure. Supervisors and EDs indeed play important roles in implementation, but frontline staff are of critical importance since they are the intervention “agents.” This finding is consistent with the CFIR, which highlights the importance of individuals’ knowledge and beliefs about the intervention as well as their competence, intellectual ability, tolerance of ambiguity, and learning style as a crucial component of the implementation system. Moreover, we have added a funder level, in addition to the community and the policy environment. The bidirectional arrows indicate considerable interaction among the ecological levels. We propose that these levels must be differentiated as indicated so that Prevention Support System addresses each level separately since the facilitators and barriers to implementation at each level are not necessarily the same.

We had theorized at the outset that greater knowledge about the intervention would be associated with greater fidelity, and this underlay our development of a thorough Prevention Support System. The manual was used extensively, with many CBO staff members calling the purple-colored manual their “Purple Bible” [34]. Yet frequently coordinators attempted to implement it without reading the manual, watching a video or attending a training, and failed to understand basic information about the MP. Knowing that many CBOs might have insufficient funds for in-person training, the manual covered all aspects of implementation and was low-cost or free if downloaded from our website. It was not only coordinators who sometimes did not understand the intervention, but many supervisors, EDs, and funders were also unaware of how the intervention worked. This resulted in poor planning, poor supervision, EDs who refused to implement some parts of the intervention, and funders who did not understand what contractual deliverables were needed. Supervisors and EDs who understood the EBI were more likely to implement it with fidelity. The variability in use of implementation materials was evident in TA sessions with coordinators. TA was provided proactively, not waiting for them to request it since our prior work had indicated that they often do not do so until they are in crisis [31,34,49]. Some TA involved explanations of basic concepts (e.g., the difference between the core group and M-groups), whereas other TA focused on more sophisticated issues, such as project adaptation or how to reach into different social networks. TA was not provided to supervisors, EDs, or funders, unless it was requested. These results suggest that it should be proactively provided to them as well.

Many of the issues that emerged correspond to concepts of organizational readiness (an inner setting component in the CFIR model), including planning for the intervention before implementation (a process domain in the CFIR), desire to change the agency’s existing prevention approach (an inner setting domain in the CFIR that is part of the implementation climate construct), belief in the efficacy of the intervention (an individual domain in the CFIR), knowledge about the intervention (an individual CFIR domain), and organizational stability (an inner setting domain in CFIR that is part of the structural characteristics construct). While these issues all relate to readiness to implement a new program, they refer to very different facets of the concept. Casteñada et al. [50] noted that readiness to implement has been a very broad conceptual category and their research review revealed four domains. While our findings could fit into these domains, recognizing the distinctiveness of the issues would likely make them easier to address in a Prevention Support System. For example, desire to change the existing prevention approach and belief in the efficacy of the EBI might be considered flip sides of the same issue, but this is not necessarily true. Many CBOs were ready to change from what they had been doing, but did not necessarily buy into the MP. Other CBOs were not dissatisfied with what they had been doing, but were happy to implement the MP. Achieving institutional buy-in and agency change are often necessary for successful implementation [42]. We found preparation to implement to be of considerable importance, and seems to be at the core of organizational readiness. But preparing front-line staff for change is quite different than convincing CBO management that change is necessary or helping a CBO determine if MP is the correct fit for its capacity and community. Addressing each issue would likely call for tailored discussions in implementation materials, TA, and training, and therefore, we have not found it useful to relegate these different issues to being “ready to implement.”

It was desired that CBOs adapt the MP for their locale and their population of YBGM after gaining an understanding of the core elements, guiding principles, and overall integrity of the intervention. Although Fixsen et al. [43,51] suggest that organizations first implement a program with fidelity and adapt a program thereafter, we have felt that that adaptation may be necessary to do from the start of implementation or it will not engender target population or agency buy-in. It has been suggested that programs should add but not delete or overly modify existing core elements [52]. This is what many CBOs did as they recognized that while the basic ideas and approaches should be preserved, it would not be problematic to add to the program, especially when done to further the social processes the program was seeking to propel (e.g., diffusion of messages or empowerment of YGBM). Indeed, many CBOs made intentional changes after a careful planning process that included analyzing if the modifications retained fidelity to MP’s guiding principles and core elements. However, there were also CBOs that drastically changed (or eliminated) core elements without considering how the intervention would work with such alterations. These changes were often unintentional, and occurred as the result of poor or no planning, or the lack of evaluation and critical analysis of the program functioning. Such changes often had deleterious effects.

The frequency with which a theme emerged was not necessarily associated with its impact on implementation success, as some issues had considerable impact even if they did not occur often. For example, funders’ refusal to pay for particular core elements was not a frequent occurrence, but when it happened, it had deleterious effects. In addition, the themes do not necessarily have independent effects, and instead can compensate for other barriers. For example, some programs had sufficient resources but poor leadership and challenging organizational issues to contend with (e.g., lack of interest in changing what they were doing) which adversely affected the program’s implementation. Other programs had highly effective staff who were able to motivate volunteers to take on important tasks and were creative in how they implemented core elements, while having low funding. Yet even the best of staff could not implement the intervention when the organization became unstable and went bankrupt.

The two most important factors affecting implementation with fidelity seemed to be resources/funding (outer setting CFIR domains), similar to others’ findings [15], and having effective frontline staff (individual CFIR domain) [43]. The MP was often underfunded, making it challenging to implement fully. Moreover, the policy change at the CDC that greatly increased HIV testing (an outer setting CFIR domain) [52] often resulted in funding for MP to be redirected to it, further reducing resources for implementation.

An important finding was the considerable variability in the extent to which CBO staff engage in reflection, analysis, hold themselves and others accountable for their work, and evaluate if they are reaching program objectives (all process CFIR process domains). These findings, in accordance with others’ recommendations [43,51,53], indicate that TA should include feedback on fidelity, especially if CBOs are not evaluating themselves. Empowerment evaluation [54] would seem particularly helpful, and might avoid a feeling of “top-down” evaluation that can put individuals into defensive modes of response.

These findings suggest that CBOs would benefit from capacity building assistance shortly after being funded, not just once they are implementing the intervention, and that it should be provided proactively instead of waiting for CBO staff to identify their needs. Although some researchers have suggested that CBOs go through specific processes when planning to implement an intervention, including a stage in which they examine their own capacities and consult with the community [9,55], we feel it would be helpful for EBI-specific capacity building to be provided to the CBO immediately upon being funded to help them make the best decisions from the start [38].

Organizational issues also need to be addressed in capacity building efforts, although the term “capacity” may suggest primarily focusing on knowledge, competencies, and skills building to the exclusion of other issues [56,57]. For example, the ED who did not trust YGBM to make decisions for the project did not necessarily lack the knowledge to run the intervention. Likewise, coordinators and supervisors who never looked at the manual or attended a training lacked knowledge of the program, but also needed to shift their attitudes regarding the importance of using such materials. Hence providing what is called “capacity building assistance” does not necessarily solely address knowledge or skills acquisition. Instead, capacity building may require attempting to change attitudes and motivations. This is in keeping with the definition of capacity that Flaspohler et al. [30] use: “the skills, motivations, knowledge, and attitudes necessary to implement innovations.” (p.183).

This research was not without its flaws. It would have been preferable to have recorded and transcribed the data for this study and to conduct site visits to the projects to observe challenges and facilitators to effective implementation firsthand. However, these were both prohibitively expensive, and so we relied on telephone interviews, extensive note-taking and participants’ description of their projects and the facilitators/challenges they experienced.

## Conclusions

Capacity building is vitally important in helping organizations in their implementation of EBIs [41,57]. The MPTES was developed to build the capacity of CBOs to translate an HIV prevention EBI into practice. These results suggest that a Prevention Support System, such as the MPTES, should address the entire ecological system in which a program occurs, ranging from the frontline staff to the broader system of HIV prevention, including the impact of national policies. In addition, capacity building should focus on bringing individuals at different levels of the implementation system into alignment regarding understanding the program’s goals and methods, including CBOs’ frontline staff, supervisors, and EDs, as well as funders, and should target helping them all to gain an in-depth understanding of the program, buy into the new approach, and plan for implementing the program before attempting to move it into practice. Moreover, capacity building should address the importance of having expectations of accountability, and seek to increase front-line staff’s motivation and skills to reflect and analyze the program’s functioning. Importantly, the issues to address with individuals at different levels of the implementation system vary considerably. Finally, since a Prevention Support System is only effective if it is actually used, it is essential to focus on how to increase the willingness of CBO staff and funders to use such a system. It must be very user friendly, including being in an acceptable format. For a Prevention Support System to be maximally useful, it must address facilitators or barriers to implementation, address the right people, and use modalities to convey information that are acceptable for users of the system.

## Abbreviations

AIDS: Acquired immune deficiency syndrome
CDC: Centers for disease control and prevention
CFIR: Consolidated framework for implementation research
D&I research: Dissemination and implementation research
DEBI project: Diffusion of effective behavioral interventions, a program by the CDC to diffuse evidence-based interventions into practice
EBI: Evidence-based intervention
ED: Executive director
HIV: Human immunodeficiency virus
M-groups: M-groups, this is not an acronym but is the name for the small group component of the Mpowerment project
MP: Mpowerment project, the intervention being studied in this research also referred to as a program
MPTES: Mpowerment project technology exchange system, our integrated system of training, ongoing technical assistance (TA), implementation materials (manuals and videos), and web-based services.
TA: Technical assistance
TRIP Study: Translating research into practice study
YGBM: Young gay or bisexual men
